# Breaking Barriers in Mental Health: Addressing Mental Health Disparities in Marginalized Communities

**DOI:** 10.3390/healthcare14172708

**Published:** 2026-08-25

**Authors:** Yok-Fong Paat

**Affiliations:** Graduate College of Social Work, University of Houston, Houston, TX 77204, USA; ypaat@uh.edu

Mental health, which encompasses emotional, psychological, and social well-being, not merely the absence of mental disorders, is a core human right [1]. It includes individuals’ ability to navigate life’s stresses, realize their potential, and contribute to their community’s well-being, making it a key pillar of human development [2]. In the United States, mental health disparities remain a widespread phenomenon, especially after the COVID-19 pandemic [3,4]. Specifically, it is estimated that more than one in five individuals in the U.S. live with a mental illness [5,6], and slightly over 60 million U.S. adults experience any mental illness [7]. However, a significant proportion of those living with mental health conditions do not seek treatment [5,8]. About 21.2 million adults have co-occurring mental illness and substance use disorder, also known as comorbidity, where the presence of multiple conditions further complicates treatment outcomes and progress [9]. Mental illness is a major contributor to the global burden of disease [10]. The World Health Organization estimated that over one billion people worldwide live with mental health disorders [11]. At both the micro and macro levels, the impacts of mental health are profound and far-reaching. At the micro level, mental illness can contribute to poor daily functioning, health outcomes, difficulties in maintaining relationships, academic and employment challenges, and reduced earning capacity and life expectancy [12,13,14,15,16]. At the macro level, mental illness can lead to substantial economic losses and societal costs, including increased healthcare and treatment expenditure, reduced workforce productivity, and increased financial strain on both the healthcare and criminal justice systems [17,18,19].

Empirical evidence indicates that mental health is closely interconnected with physical health outcomes and overall daily functioning [16,20]. Nevertheless, the burden of mental and physical illness is unevenly distributed, with disadvantaged groups experiencing poorer health outcomes from birth and throughout their lives [21,22]. In the U.S., health disparities are widening, particularly among economically disadvantaged and socially marginalized populations, reflecting patterns of inequity observed in health outcomes globally [23,24]. Social determinants of health are non-medical factors shaped by broader social and economic conditions that influence well-being. They include the circumstances in which people are born, grow, live, work, and age, and they play a central role in shaping health inequities [21,22]. Many of these disparities are avoidable and are often driven, in part, by unequal access to quality healthcare [22,25]. Strengthening mental health and prioritizing equity by addressing social, economic, and systemic barriers are key to reducing systemic disparities in care quality and health outcomes faced by underserved communities [26]. To this end, promoting health equity ensures that everyone has a fair and just opportunity to attain optimal health. Marginalized communities consist of vulnerable and at-risk populations, including low-income groups, racial and ethnic minorities, immigrants, and refugees, who often bear a disproportionate burden of challenges linked to chronic stress, structural inequality, poverty, and trauma [27,28]. As a result, these populations frequently experience higher rates of mental health challenges driven by the combined effects of discrimination, violence, stigma, and ongoing socioeconomic disadvantages, which increase their risks for depression, anxiety, and mental health concerns [24].

While treatment may contribute to improved mental health outcomes, at-risk populations must confront structural barriers to accessing culturally and linguistically competent mental health care and services, which can perpetuate the cycle of marginalization [29]. Addressing social determinants of mental health can reduce systemic barriers to care. Individuals may experience discrimination based on socioeconomic status, religion, gender, disability, or sexual orientation, which is often associated with poorer health outcomes. These disparities are further reinforced by provider and systemic bias, as well as barriers such as insurance costs and unequal access to care [30,31]. In addition, poverty-related factors such as food insecurity and housing instability are strongly linked to poorer physical and mental health. Chronic health conditions, in turn, can further compound this burden, increasing the risk of mental health conditions such as depression and anxiety [32,33]. In their article in this Special Issue, Figueroa and colleagues analyzed a U.S. census-matched sample and, using mediation models, found that perceived stress statistically mediates the relationship between classism and mental health symptoms, highlighting perceived stress as a potential pathway linking classism to mental health symptoms [34]. In the article by Paat and colleagues, the authors examine the mental health challenges and barriers that hinder veterans’ adjustment to civilian life along the U.S.–Mexico border—an underserved region with limited access to care—through semi-structured interviews and surveys with 36 veterans recruited from a mental health agency in Southwestern Texas. They found that participants faced persistent difficulties related to mental health, the transition from military to civilian life, strained relationships, discrimination, and limited access to opportunities and services [35]. By deepening our understanding of marginalized communities and implementing culturally responsive mental health practices, we can strengthen community well-being.

Racial and ethnic minorities are also more likely to experience severe and persistent mental health conditions, while facing limited access to adequate care as well as a broad range of barriers to mental health services [36]. Research evidence suggests that African Americans and other racial minorities are disproportionately exposed to social and environmental conditions, such as community violence, which can adversely affect mental health [37]. In addition, they face a comparatively higher burden of mental illness and experience disparities in assessment, treatment, and access to supportive services. Black and ethnic minority groups, in particular, are less likely to receive treatment from mental health professionals [26,36,38]. Ethnic and cultural bias can also interfere with the accurate diagnosis of mental health conditions. Certain personality disorders, in particular, are more frequently diagnosed among Black patients. For example, Blacks are more likely to be diagnosed with schizophrenia and to be admitted to hospital for its treatment [39]. Unfortunately, many individuals do not seek treatment for their mental health condition or do not receive adequate care. Varying cultural beliefs about mental health, as well as longstanding traditional stereotypes, can act as barriers that discourage individuals from seeking support [40,41]. Furthermore, racial minorities are more likely to present with physical or somatic concerns rather than mental health symptoms. Research also suggests that Black and ethnic minority groups are more likely to receive mental health support through primary care and emergency care when accessing treatment [42,43,44]. Additionally, racial and ethnic minorities are more likely to suffer from severe and persistent disability due to mental illness [36,45]. They are also disproportionately more likely to come into contact with the criminal justice system, when many of these situations would be better addressed through timely and appropriate mental health care and support [46]. In her article, Parker examined mental health disparities among Black mothers through interviews. She found that Black mothers tend to prefer culturally congruent, accessible, and stress-reducing forms of support. Their experiences are shaped by their identities and past experiences, highlighting the need for nonjudgmental, culturally responsive, and strengths-based care to advance mental health equity [47].

Next, acculturative attitudes can also shape mental health care and outcomes [48,49,50]. Immigrants may migrate for economic, political, or cultural reasons and are often at increased risk of poor psychological health outcomes due to limited resources, restricted economic opportunities, and stigma [51]. They may also face barriers to accessing care, such as limited availability of interpreters and low awareness of available services. Refugees often come from countries affected by civil unrest and armed conflict. Asylum seekers and refugees, in particular, are at higher risk of poor mental health outcomes due to exposure to trauma and violence [52,53,54]. Rape and sexual violence are also common contributors to post-traumatic stress disorder [55,56]. Indeed, Adedeji and colleagues examined the mental health outcomes and coping strategies of 15 female Ukrainian refugees living in Germany during displacement and resettlement. Their mixed-methods study found that participants experienced depression, isolation, disconnection, cultural adjustment difficulties, and psychological distress linked to war, displacement, migration, and uncertainty. However, the study also highlighted coping strategies employed to mitigate these mental health challenges, including staying active, volunteering, and seeking community support, underscoring their resilience [57].

Understanding the risk and protective factors that shape child mental health is especially critical, as childhood experiences often influence mental health outcomes across the life course into adulthood [58,59]. Morgan and colleagues explored the experiences of adult survivors of childhood sexual trauma in South Africa using culturally grounded and trauma-informed approaches. Interviews with survivors from underserved communities revealed that healing is often not straightforward, highlighting the importance of culturally responsive care, relational safety, somatic and embodied approaches in trauma recovery, as well as the need for more inclusive, community-based support networks [60]. Lin’s research examines emotional self-efficacy as a mediator between positive leisure experiences and subjective well-being among elementary school-aged children in a marginalized U.S.–Mexico border community in Texas. Her findings suggest that positive leisure experiences enhance children’s well-being by strengthening emotional self-efficacy [61].

Taken together, there is a need for more community- and policy-level research aimed at reducing mental health disparities and identifying effective practices that address these inequities [43]. Access to timely and appropriate mental health care can play a preventive role in supporting individuals confronting persistent mental health challenges [62,63]. Improving health equity requires efforts to enhance health outcomes for those facing social and economic disadvantages. Some researchers have criticized Eurocentric models of recovery for Black and ethnic minority groups, arguing that they overlook issues of racial inequality and fail to account for the broader social factors that shape individuals’ experiences [64,65]. Others suggest that aligning the cultural, linguistic, and racial identities of providers and patients can improve treatment outcomes among ethnic minority groups and support recovery [66,67]. Practitioners need to be aware of and responsive to the cultural and faith beliefs of Black and ethnic minority populations, as understanding these beliefs and adopting approaches that incorporate mental health, physical health, culture, and belief systems can help support recovery [66]. Healthcare providers have a responsibility to recognize and address the social determinants that shape the physical and mental health of vulnerable populations [68], including addressing the need for culturally adapted interventions.

In their article in this Special Issue, Mallonee and colleagues developed and pilot-tested *Pensamientos y Pláticas*, a four-week closed, community-grounded mental health engagement program serving Hispanic communities in the El Paso region in Texas. They found a significant reduction in stigma related to help-seeking, supporting the program as a promising culturally responsive intervention for stigma reduction [69]. Reducing health disparities entails improving community health and well-being, establishing mutually beneficial partnerships and collaborations, and pooling diverse expertise, skills, and knowledge to solve health problems and promote health equity [70]. Global health interventions extend beyond disease control to tackle broader social, economic, cultural, and contextual factors that impact health outcomes [22,70]. Yesha and colleagues conducted a narrative review examining the use of AI in digital mental health and its implications for marginalized communities. They also emphasize the importance of an intersectional, culturally responsive approach to developing equitable digital mental health tools that reflect lived experiences and minimize harm [71]. Successful implementation requires sustained effort and strong coordination across government sectors. It also depends on ensuring access to resources that support healthy behaviors, reduce environmental risks, and strengthen individuals’ capacity to achieve optimal health [72]. In sum, addressing poor mental health outcomes will therefore require a shift in priorities toward preventing or reducing the social conditions that undermine health, alongside strengthening evidence-based policies and interventions.
